# Study protocol for prospective multi-institutional phase III trial of standard of care therapy with or without stereotactic ablative radiation therapy for recurrent ovarian cancer (SABR-ROC)

**DOI:** 10.1186/s12885-023-11407-y

**Published:** 2023-10-20

**Authors:** Yong Bae Kim, Hwa Kyung Byun, Chan Woo Wee, Hojin Kim, Seyoung Kim, Gowoon Yang, Jina Kim, Sang Joon Park, Jung-Yun Lee

**Affiliations:** Yonsei University College of Medicine, 50-1, Yonsei-ro, Seodaemun-gu, 03722 Seoul, Republic of Korea

**Keywords:** Recurrent ovarian cancer, SABR, Overall survival

## Abstract

**Background:**

Efforts have been made to investigate the role of salvage radiotherapy (RT) in treating recurrent ovarian cancer (ROC). Stereotactic ablative radiation therapy (SABR) is a state-of-the-art therapy that uses intensity modulation to increase the fractional dose, decrease the number of fractions, and target tumors with high precision.

**Methods:**

The SABR-ROC trial is a phase 3, multicenter, randomized, prospective study to evaluate whether the addition of SABR to the standard of care significantly improves the 3-year overall survival (OS) of patients with ROC. Patients who have completed the standard treatment for primary epithelial ovarian cancer are eligible. In addition, patients with number of metastases ≤ 10 and maximum diameter of each metastatic site of gross tumor ≤ 5 cm are allowed. Randomization will be stratified by (1) No. of the following clinical factors met, platinum sensitivity, absence of ascites, normal level of CA125, and ECOG performance status of 0–1; 0–3 vs. 4; (2) site of recurrence; with vs. without lymph nodes; and (3) PARP inhibitor; use vs. non-use. The target number of patients to be enrolled in this study is 270. Participants will be randomized in a 1:2 ratio. Participants in Arm 2 will receive SABR for recurrent lesions clearly identified in imaging tests as well as the standard of care (Arm 1) based on treatment guidelines and decisions made in multidisciplinary discussions. The RT fraction number can range from 1 to 10, and the accepted dose range is 16–45 Gy. The RT Quality Assurance (QA) program consists of a three-tiered system: general credentialing, trial-specific credentialing, and individual case reviews.

**Discussion:**

SABR appears to be preferable as it does not interfere with the schedule of systemic treatment by minimizing the elapsed days of RT. The synergistic effect between systemic treatment and SABR is expected to reduce the tumor burden by eradicating gross tumors identified through imaging with SABR and controlling microscopic cancer with systemic treatment. It might also be beneficial for quality-of-life preservation in older adults or heavily treated patients.

**Trial registration:**

This trial was registered at ClinicalTrials.gov (NCT05444270) on June 29th, 2022.

**Supplementary Information:**

The online version contains supplementary material available at 10.1186/s12885-023-11407-y.

## Background

Ovarian cancer is the third most common gynecological cancer in Korea and its incidence has gradually increased. As of 2017, 2,505 women were newly diagnosed with ovarian cancer annually. Advanced ovarian cancer is known to have the worst treatment outcomes among gynecologic cancers. The treatment of choice for advanced ovarian cancer is maximal debulking surgery followed by adjuvant platinum-based chemotherapy. Because epithelial ovarian cancer is generally detected at an advanced stage, many patients experience recurrence after standard first-line treatment. Despite repeated salvage chemotherapy, the recurrence interval becomes shorter, and the 5-year survival rate is the lowest among gynecologic cancers at less than 40–50% [1].

Radiation sensitivity has been widely reported in patients with epithelial ovarian cancer. After the publication of McGuire’s study, which demonstrated the efficacy of paclitaxel and cisplatin, radiation therapy lost its prominence as an adjuvant therapy for epithelial ovarian cancer [2]. Efforts have been made to investigate the role of salvage radiotherapy (RT). Among retrospective studies, the M.D. Anderson Cancer Center study, recognized as a landmark publication, eloquently highlighted the pivotal role of involved field RT (IFRT) in the management of recurrent ovarian cancer (ROC), shedding light on the critical influence of lymph nodes, clear cell carcinoma, and platinum sensitivity on treatment efficacy and patient outcomes [3]. A phase 2 prospective clinical study, Korean Radiation Oncology Group (KROG) 14 − 05, reported high local control and acceptable safety of IFRT in 30 patients. Despite the failure to meet the primary endpoint, which was attributed to the prevalence of out-field recurrence as the primary pattern of treatment failure, an interesting finding emerged. In certain cases of repeated recurrence, it was possible to implement a drug holiday strategy, wherein patients received exclusive treatment with IFRT without the administration of chemotherapy [4].

Stereotactic ablative radiotherapy (SABR) is the latest radiation therapy technique that uses intensity modulation to increase a single dose, decrease the number of fractions, and target tumors with high precision. In the SABR-COMET study, patients with oligometastases of breast, lung, colorectal, and prostate cancer were randomized to palliative standard of care and standard of care plus SABR groups, and the overall survival (OS) rate was significantly increased in patients with the addition of SABR [5]. In addition, three retrospective studies have reported outstanding treatment efficacy and a favorable safety profile for SABR [6–8]. Consequently, it is imperative to conduct a prospective trial to substantiate the role of SABR in the management of ROC.

## Methods and design

### Study design

**Fig. 1:**
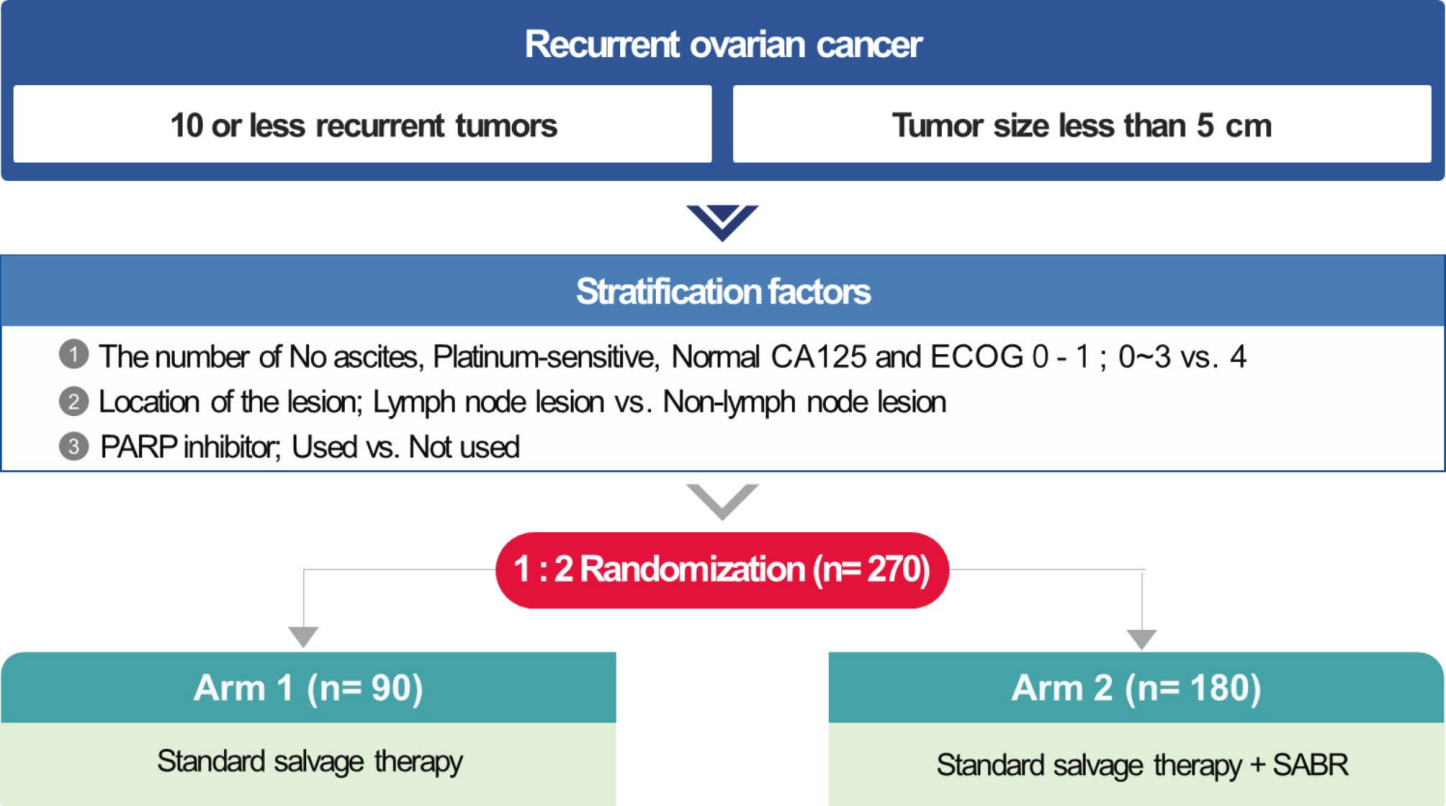
Study scheme

This is a phase 3, multicenter, randomized, prospective study to evaluate whether the addition of SABR to the standard of care therapy significantly improves the 3-year overall OS in patients with ROC. Participants are stratified according to the following criteria before randomization: (1) platinum sensitivity, absence of ascites, normal level of CA125, ECOG performance status of 0–1; 0–3 vs. 4; (2) site of lymph node recurrence: no vs. yes; and (3) PARP inhibitor use vs. non-use. This study randomly assigns the participants to Arm 1 and Arm 2 at a ratio of 1:2 (Fig. 1). Participants assigned to Arm 1 will continue to receive the current salvage therapy suitable for individuals by considering factors such as the location and size of recurrence, and the patient’s comorbidities, at the discretion of the physician. Patients are recommended to receive standard care therapy according to the National Comprehensive Cancer Center (NCCN) guidelines for ROC. Participants in Arm 2 will undergo SABR for recurrent lesions that are clearly identified on routine imaging tests. After SABR, additional standards of care therapy will be continued as planned at the discretion of the physician. Additional salvage therapy will continue before or after SABR, as planned, at the discretion of the physician. After SABR is performed on recurrent lesions identified at the time of registration, additional SABR may be performed on newly developed recurrent lesions during follow-up at the physician’s discretion (no limit on the number of times). This trial is anticipated to run for 5 years, with a 2-year enrollment phase and a 3-year follow-up. The first participant was recruited in November 2022. Participant registration is expected to be completed by December 2024, and the final results will be available after 2027. This protocol has been approved by the Korean Gynecologic Oncology Group (KGOG) and KROG and has been designated as KGOG 3064 and KROG 2204. This trial was registered at ClinicalTrials.gov (NCT05444270) on June 29, 2022.

### Eligibility criteria

Patients who meet all of the following criteria will be included in this study: (1) pathologically proven epithelial ovarian cancer; (2) patients must have completed treatment for the primary tumor (maximal debulking operation and adjuvant platinum-based chemotherapy according to the stage); (3) number of metastases allowed: ≤10 (adjacent lesions can be counted as a single lesion if possible to be included in a single RT treatment plan; left cervical lymph node, right cervical lymph node, center of the left lung, periphery of the left lung, left pleura, center of the right lung, periphery of the right lung, right pleura, mediastinal lymph node, left lobe of the liver, right lobe of the liver, perihepatic space, spleen, perisplenic space, within two vertebrae above or below based on the spine with the lesion, abdominal cavity, pelvic cavity, paraaortic lymph node, and pelvic lymph nodes); (4) maximum diameter of each metastatic site of gross tumor ≤ 5 cm; (5) age ≥ 19 years old; (6) sufficient bone marrow function on tests performed within 60 days of registration; (7) Zubrod Performance Status Score ≤ 2 at the time of registration; and (8) patients must submit the informed consent form related to the study before participating in the study.

Patients who meet any of the following criteria will be excluded from this study: (1) brain metastasis; (2) diffuse peritoneal carcinomatosis; (3) exudative, bloody, or cytologically proven malignant pleural or pericardial effusion; (4) a history of RT; (5) lesions unsuitable for targeting because of unclear borders; (6) co-existing or underlying invasive cancer (excluding thyroid cancer, cervical CIS, basal cell carcinoma of skin, and early gastric cancer) that has not achieved disease-free status for ≥ 3 years; and (7) serious comorbidities.

### Sample size calculation

The supporting data for calculating the number of participants required for this study were based on the results of a retrospective analysis of the treatment results of patients with ROC at the Yonsei Cancer Center (unpublished data). In this analysis, the 3-year survival rates were 74.4% and 58.5% in the IFRT and non-radiation treatment groups, respectively. Based on this, the type I error rate was set at 0.05; statistical power, 80%; 1-year dropout, 5%; randomization ratio, 1:2; accrual time, 2 years; and follow-up, 3 years. As a result, the number of patients needed was calculated as 90 for Arm 1 and 180 for Arm 2.

### Obtaining consent and screening procedures

Patients eligible for enrollment are considered through multidisciplinary discussions with oncologists. The investigator explains the background, purpose, and procedures of this study to the patients and their guardians and gives them sufficient time to decide whether to participate in the study. If the patient agrees to participate in the study, informed consent is obtained. Patients are informed that they can indicate their intention to withdraw from the study at any time and that their participation in the study will be immediately discontinued upon withdrawal. To minimize the possibility of coercion or undue influence, the investigators explain that the research patient’s decision to participate in the research is voluntary, they can refuse to participate in the research or withdraw participation at any time during the research period, and there will be no disadvantages for the next treatment.

The following screening tests must be performed to for patients to participate in this clinical trial: clinical examination, history taking and physical examination, complete blood count (once, routine test volume), routine chemistry to evaluate hepatic and renal function (once, routine test volume), computed tomography (CT) or positron emission tomography (PET)-CT to confirm the size and location of the recurrent lesion, and tumor marker CA125. However, these need not be conducted if they have been performed within 2 months of registration.

### SABR

This study requires only a photon beam of 6 MV or more: both intensity modulated RT (IMRT) and volumetric modulated arc therapy with linear accelerators are acceptable, and TomoTherapy (Madison, WI, USA) and CyberKnife (Sunnyvale, CA, USA) can be used. All participants should have a CT-based treatment plan, and CT slices should be 3 mm apart or smaller, if necessary. Multiple CT scans are also permitted if there are two different treatment sites such that images cannot be obtained simultaneously. It is recommended that all treatment sites be targeted with the patient in a single treatment position. However, the patient’s position can be altered when RT is administered to the lungs and extremities. All lesions that may move during breathing should be evaluated using 4D CT, fiducial markers, or fluoroscopy. In addition, for lesions with movement of ≥ 5 mm, breathing control techniques, including the use of an abdominal compression device, active breathing control, breathing suppression, gating, and tracking, or internal target volume techniques, are all recommended. All treatments in this study require an image guidance technique that can identify the target of treatment in three perpendicular directions (superior/inferior, left/right, and anterior/posterior). Cone Beam CT, Mega-Voltage CT, dual fixed-position in-room kV imaging system, in-room diagnostic CT, and TomoTherapy approaches are all accepted. Target volumes are defined as follows: The gross tumor volume (GTV) includes all recurrent sites observed on planning CT and additional PET-CT. The clinical target volume is not defined due to the nature of this study. To account for setup error and movement of internal organs, a margin of 3–5 mm around the GTV is set to generate the planning target volume (PTV). Multileaf collimator shielding is used based on the PTV.

The recommended dose fractions are listed in Table 1. In the case of 6–9 fractions, the prescribed doses of 5 and 10 fractions, along with dose constraints, are applied proportionally. The prescription isodose surface is selected to include 90% of the PTV on the prescription isodose curve. Doses less than 90% of the prescription dose are limited to the outer boundary of the PTV. As an exception, 70% coverage is allowed for some PTVs if small lesions are collectively formed and have irregular shapes. However, maximum efforts should be made to ensure that the GTV is covered by 100% of the prescribed isodose surface. The prescription isodose surface should be determined from 60 to 100% of the maximum dose in the PTV. The maximum dose should be 100–166.67%, based on the prescription isodose surface, and is normalized to 100%. If a considerable volume of OARs is included in the PTV, a treatment plan using 10 fractions is recommended, and in this case, PTV-EVAL can be set separately to evaluate the PTV coverage. PTV-EVAL is defined as the remaining part of the predefined PTVs, excluding OARs (bowel, duodenum, rectum, bladder, etc.) during treatment planning. However, in this case, even if the evaluation of the target is based on PTV-EVAL, the minimum dose of the PTV should be ≥ 70% of the prescribed dose. If it is difficult to achieve the R_50%_ conditions, excluding the evaluation of the ratio of the prescription isodose volume, it is evaluated as an acceptable treatment plan, even if it is not met. However, the R_50%_ and D_2cm_ are evaluated using the existing PTV, and lowering the OAR dose should be prioritized over dose fall-off in this case. When a treatment plan with PTV-EVAL is evaluated, prior consultation should be communicated to the research headquarters (Yonsei Cancer Center, Seoul, South Korea) to prevent decision as an unacceptable deviation.

**Table 1:** Recommendation for dose fractionation of SABR

No. of Fraction	Representative Dose (Gy)	Acceptable Dose (Gy)
1	20	16–24
3	30	24–33
5	35	25–40
10	40	35–45

### Safety monitoring and follow‑up

Adverse events to be recorded include hematological, gastrointestinal, renal/genitourinary, dermatological, and other adverse effects and will be evaluated based on the Common Terminology Criteria for Adverse Events (CTCAE) version 5. An institutional investigator will report all serious adverse events to the institutional review board of each participating center and the primary investigator.

After the completion of RT, observation and clinical examination will be conducted as follows: interview with study staff at 3, 6, 9, 12, 18, 24, 30, and 36 months after registration, history taking, physical examination, and evaluation of all possible side effects. Related tests can be prescribed if necessary, imaging tests (either CT or PET-CT) including the treatment site or suspected recurrence area, tumor marker CA125 test at every visit after registration, and survey (using the EORTC quality of life questionnaire (QLQ)-C30 questionnaire before randomization, and at 12, 24, and 36 months after registration for all participants; for participants in Arm 2, Cancer Therapy Satisfaction Questionnaire (CTSQ) is also used before randomization, and at 12, 24, and 36 months after registration).

### Data management

The principal investigator is responsible for managing the clinical trial data. Investigators will complete the case report forms using a web-based system (https://www.mytrial.co.kr/). Data monitoring will be performed by a clinical research organization (KGOG, Seoul, South Korea) and the clinical research coordinator of each participating center. Documents generated from this trial will be stored at each participating center for 10 years after trial completion with the approval of the institutional review board, after which personal information will be discarded.

For patients who agree to participate, 20 unstained slides are sectioned from formalin-fixed paraffin-embedded blocks of tumor tissue during the primary debulking operation in participating institutions. The slides are sent to research headquarters for next-generation sequencing. Tumor samples already stored at each institution are collected for genome analysis, and there is no plan to acquire additional tissue for this study. Except for the 20 slides, tissue blocks will be stored in the pathology department of each institution, and the research headquarters will keep them for up to five years after genomic analysis.

To develop a patient selection and prediction model in which SABR can improve clinical outcomes, artificial intelligence (AI)-based radiomics analysis is performed to predict the molecular subtype using the patient’s CT and/or PET-CT images. Imaging data are collected from each institution and transferred to the research headquarters for analysis.

### Endpoint and statistics

The primary endpoint is OS (failure or death from any cause). Secondary endpoints include control of existing recurrence sites, new recurrences, adverse events, chemotherapy-free intervals, health-related quality of life, discovery of treatment-refractory molecular subtypes through next-generation sequencing, development of a prediction model using AI-based radiomics, and analysis of other clinically meaningful descriptive variables described in the protocol.

The primary hypothesis of this study is that the addition of SABR to the standard of care therapy in patients with ROC would improve the OS from 58.5 to 74.42%, corresponding to a hazard ratio of 0.55. Survival is estimated by Kaplan–Maier method, and survival failure is defined as the subject’s death from any cause. The log-rank test is used to compare the distribution of survival estimates between the Arm 1 and Arm 2 participants. The survival period is defined as the period from the date of randomization to the date of death. Imaging tests are performed every 3 months for the 1st year, every 6 months for 3 years, and every 6 months thereafter until disease progression. The Cox proportional hazards regression model is used to analyze factors affecting the OS along with treatment methods.

## Discussion

IFRT has recently been used as a salvage treatment for recurrent cancer in cases where RT has not played a role in ovarian epithelial cell carcinoma for a significant period. According to reports from various institutions, IFRT has shown good results in terms of tumor control and has acceptable side effects [3, 9, 10]. However, because IFRT requires a treatment period of approximately 5 weeks, cytotoxic chemotherapy cannot be administered during this period. Therefore, the main pattern of relapse after IFRT occurs when the disease progresses beyond the radiation field. Hence, relapse can be prevented by minimizing the number of elapsed days of RT to allow cytotoxic chemotherapy to fit into the schedule.

Therefore, SABR could be an alternative to minimize these issues. Because IFRT includes adjacent microscopic disease, but SABR only covers gross tumors, it minimizes the treatment period by increasing the fractional dose. If the fractional dose is extremely high, setting up considering high-precision treatment is required from the simulation room to the treatment room. This is because the treatment technique is based on IMRT, and the error should be minimized using image-guided RT techniques. The expected clinical benefits of SABR are as follows: First, SABR performs high-precision treatment within five fractions per 10 business days. Additionally, it does not interfere with the chemotherapy schedule and will not result in deterioration of the physical condition. Second, its high fractional dose can be used to treat radiation-resistant tumors that are not controlled by conventional fractionation. Third, because it minimizes the irradiated dose to the surrounding normal tissue, re-irradiation may be easier when true or marginal recurrence occurs. Fourth, in cases where cytotoxic chemotherapy and targeted therapy are used as maintenance therapy, effective anticancer drugs can be continued after treating only some progressive lesions with SABR, rather than stopping or changing drugs when tumors show heterogeneous responses.

The implementation of this three-tiered RTQA program ensured adherence to standardized protocols and increased the precision and reliability of SABR across different institutions and radiation oncologists. By establishing and following this comprehensive program, the quality of SABR treatment can be effectively monitored and improved, leading to better patient outcomes and enhanced credibility of the clinical research in the field of radiation oncology. In the current study, a three-tiered RTQA program is devised. The first tier involves conducting a survey among the participating institutions to assess their faculty and equipment capabilities in performing SABR. Additionally, a certification program is completed to verify the basic mechanical and dosimetric accuracy of the SABR equipment and any deficiencies are addressed. In the second tier, participating researchers are provided with dummy cases for SABR treatment as examples. They define the contours of the target and organs at risk and develop treatment plans based on the dose-volume constraints outlined in the protocol. The research headquarter evaluates the plans and provides feedback. Furthermore, advanced dosimetry checks are conducted, including mechanical and motion management QA for each item of equipment used in the SABR treatment. In addition, phantom studies are conducted to ensure accuracy. The third tier involves individual case reviews. The first SABR plan from each institution should be sent at least 48 h in advance to research headquarter. Treatment decisions are made only after confirming that the dose-volume constraints are met. Thereafter, the cases are reviewed sequentially.

This is a phase 3 study with 1:2 randomization. For participants assigned to Arm 1, only palliative RT is allowed for symptomatic relief, and SABR is not applicable. Although RT is not yet a standard treatment with sufficient evidence to be listed in the guidelines, it is known to be effective based on retrospective reports or clinical experiences. Therefore, gynecological oncologists are hesitant to enroll patients with oligometastatic lesions, even though RT may not be possible in only one-third of these cases. Therefore, while treatable oligometastatic cases are not well-registered, cases with a wider range of recurrent lesions, or difficult-to-treat cases that are not usually referred for RT, are registered. In particular, there is an increasing number of cases registered with peritoneal seeding, which are not indicated for SABR; therefore, a protocol was prepared to apply IFRT with 10 fractions rather than SABR in these cases.

In conclusion, the SABR-ROC study was conceived based on the current interest in determining the role of RT in ovarian cancer. With the development of RT technology, a design was developed to apply the latest technology, SABR therapy, to relapsed patients. As the main treatment for ROC is systemic therapy, in this study we are attempting to make use of the advantages of RT, which removes a limited range of gross tumors in the shortest number of days without interfering with the systemic treatment schedule. In addition, considering that the recurrence pattern of ROC differs from that of other solid cancers, the endpoint is to set a 3-year OS to verify the clinical effectiveness of RT, with the difference in survival rate depending on the presence or absence of RT.

### Electronic supplementary material

Below is the link to the electronic supplementary material.

Supplementary Material 1

## Data Availability

Not applicable.

## List of abbreviations

AI: artificial intelligence
CA125: cancer antigen 125
CT: computed tomography
CTCAE: Common Terminology Criteria for Adverse Events
CTSQ: Cancer Therapy Satisfaction Questionnaire
D_2cm_: maximum dose at 2 cm from PTV in any direction
ECOG: Eastern Cooperative Oncology Group
GTV: gross tumor volume
IFRT: involved field radiation therapy
IMRT: intensity-modulated radiation therapy
KGOG: Korean Gynecologic Oncology Group
KROG: Korean Radiation Oncology Group
NCCN: National Comprehensive Cancer Center
OARs: organs at risk
OS: overall survival
PARP: Poly (ADP-ribose) polymerase
PET-CT: positron emission tomography-computed tomography
PTV: planning target volume
PTV-EVAL: PTV for evaluation
QA: quality assurance
QLQ: quality of life questionnaire
R50%: ratio of volume of 50% prescription isodose volume to the volume of PTV
ROC: recurrent ovarian cancer
RT: radiotherapy
RTQA: radiotherapy quality assurance
SABR: stereotactic ablative radiation therapy
